# Examining mindfulness and moral disengagement in doping: Perspective of Turkish wrestlers

**DOI:** 10.3389/fpsyg.2023.1142343

**Published:** 2023-03-28

**Authors:** Erdem Ayyildiz, Hamza Kayabeşler, Mehmet Gülü, Fatma Hilal Yagin, Monira I. Aldhahi, Elena García-Grimau, Sameer Badri Al-Mhanna

**Affiliations:** ^1^Sports Science Faculty, Tekirdağ Namik Kemal University, Tekirdağ, Türkiye; ^2^Sports Science Faculty, Pamukkale University, Denizli, Türkiye; ^3^Department of Coaching Education, Faculty of Sport Sciences, Kirikkale University, Kirikkale, Türkiye; ^4^Department of Biostatistics and Medical Informatics, Faculty of Medicine, Inonu University, Malatya, Türkiye; ^5^Department of Rehabilitation Sciences, College of Health and Rehabilitation Sciences, Princess Nourah bint Abdulrahman University, Riyadh, Saudi Arabia; ^6^Department of Physical Education, Sport, and Human Movement, Universidad Autónoma de Madrid, Madrid, Spain; ^7^Department of Physiology, School of Medical Sciences, Universiti Sains Malaysia, Kubang Kerian, Kelantan, Malaysia

**Keywords:** adolescent, sports psychology, sports sociology, wrestling, doping substances, moral disengagement, mindfulness

## Abstract

**Introduction:**

Studies related to attitudes toward the use of prohibited substances in Turkish athletes are scarce. The World Anti-Doping Agency (WADA) has implemented anti-doping educational policies emphasizing doping-related education in studies conducted among Turkish wrestlers. However, it is still unclear the extent to which the wrestlers comply and adhere to these anti-doping policies. No research has previously examined the effect of anti-doping education on athletes' mindfulness and moral disengagement in doping (MDD). Therefore, the present study has a two-fold objective: first, to examine whether doping-related education (DRE) and the status of being a national athlete (NA) have an effect on athlete mindfulness and MDD. Second, to analyze the relationship between each sub-dimensions of athlete mindfulness: awareness (ASD), judgment (JSD), and refocus (RSD) with MDD.

**Methods:**

A total of 409 male wrestlers participated in this study. MANOVA analysis showed that NA and DRE alone have no effect on MDD but have a general effect on mindfulness.

**Results:**

The highest effect was on the ASD of being an NA ($\eta_{p}^{2}$ = *0*.173). When the interaction effect of NA^*^DRE was examined, significant difference in MDD (*F* = 8.218, *p* = 0.004), ASD (*F* = 8.476, *p* = 0.004), JSD (*F* = 5.844, *p* = 0.016), and RSD (*F* = 11.476, *p* = 0.001) were found. MDD has a weak negative relationship with ASD (*r* = −0.126) and RSD (*r* = −0.041) and a weak positive relationship with the JSD sub-dimension (*r* = 0.140). Those results suggest that being a NA and having received anti-doping education affect moral disengagement in doping and athletes' mindfulness.

**Discussion:**

As a conclusion, it is recommended to increase awareness and anti-doping education among national-standard Turkish wrestlers to prevent them from engaging in doping behaviors.

## 1. Introduction

Mindfulness is a psychological process that progresses by avoiding judgments and being aware of the individual's experiences in the present (Amemiya and Sakairi, 2020; Zhang et al., 2021). All athletes' behaviors to increase their performance are conscious behaviors and related to mindfulness (Henriksen et al., 2019). Mindfulness plays a vital role in determining how willing athletes are to use performance-enhancing substances (Kaufman et al., 2018). Unfortunately, the use of prohibited substances to improve performance, also known as doping, is common in sports. This situation has caused serious concern among the public. This concern led to the establishment of the World Anti-Doping Agency (WADA) in November 1999. Since that date, WADA has initiated studies to detect doping usage in sports. Although there are many physiological studies in this field, sociological and psychological studies are very few. The mindfulness of the athletes is essential in the use of doping (Tandon et al., 2015; Nolte et al., 2016). Research on mindfulness in sports has increased in the last decade due to its influence on athletic performance (Gardner and Moore, 2012; Noetel et al., 2019).

The intent of using doping is to develop endurance on the road to success, increase muscle mass and strength, delay the fatigue that will occur during exercise, and accelerate recovery after training and competition (Ersoy, 1995; Ismaili et al., 2013). In addition, doping is a heterogeneous phenomenon involving many personal, situational, and social factors (García-Grimau et al., 2021). The increasing use of doping among young people can be seen as an essential sociological problem (Striegel et al., 2006), especially the use of doping in sports among wrestling (Ehrnborg and Rosén, 2009). The normalization of the use of doping by people who are respected in society is among the most important elements of the sociological problem (Lucidi et al., 2004, 2008). Despite the viscous effect of using doping on health, it is commonly used among athletes. In the report by WADA, 3,749 wrestlers were tested, and 41 wrestlers (1.1%) tested positive in the Adverse Analytical Findings conducted on wrestlers in 2020. It is seen that this rate is below 0.5% in many Olympic sports (2020). While WADA reports rates of ~1–2%, social science studies argue that there is more doping use (WADA, 2020). The nuance perspective on doping use is extremely important (Backhouse et al., 2009a).

It should be known that athletes may consume doping substances unconsciously (Chan et al., 2015; Karatas and Dogan, 2021). For example, the Dutch anti-doping organization found that 25 (38%) of 66 food supplements were purchased online (Duiven and De Hon, 2012), and 17 (16.5%) of 103 online purchases in the US were prohibited by WADA (Baume et al., 2006). In addition, many athletes used doping with the advice of their friends and trainers (Bloodworth and McNamee, 2010; Patterson et al., 2014; Willick et al., 2016; Engelberg et al., 2019). Therefore, sports scientists should support the provision of a safe environment by conducting studies on the use of doping by athletes (Chan et al., 2015; Gülü and Yapici, 2022). WADA is constantly working on training and deterring athletes to combat doping (Chan et al., 2015; Patterson et al., 2019). WADA has declared that education is a cornerstone of anti-doping strategies. In 2021, WADA amended article 18 of the World Anti-Doping Code, and an online e-learning program was created. Yet, further initiative is required as WADA has not been successful enough in increasing the level of doping-related training of athletes (Woolf, 2020).

In the studies conducted on Turkish wrestlers, it has been emphasized that doping among Turkish wrestlers is common and that doping use should be reduced by raising awareness about doping (Gençtürk et al., 2009; Tonga, 2015; Samar and Cuma, 2022). WADA is constantly working on deterrence training for athletes to combat doping (Willick et al., 2016; Patterson et al., 2019). Thus, morals are taught in order to play a role in modulating doping behavior. The conceptual framework of the social cognitive theory of moral thought and action emphasizes that personal factors, including morality, interplay with affective self-reaction and sociopsychological determinants, which contribute to the moral reasoning of an action (Bandura, 2014). It reflects the awareness that individuals are doing something wrong when they act in violation of their ethical standards. In order to have this awareness, the athletes must have received the necessary education (Duiven and De Hon, 2012). In addition, individuals' mindfulness levels can be important in a sports context (Gardner-Nix, 2009; Röthlin et al., 2016). Such initiative to increase awareness and educate athletes may impact their perception of doping immorality and mindfulness. Therefore, the overarching aims of this study are 2-fold: First, to examine whether anti-education status and being a national athlete (NA) have an effect on athlete mindfulness and moral disengagement in doping (MDD). Second, to analyze the relationship between each sub-dimensions of athlete mindfulness: awareness (ASD), judgment (JSD), and refocus (RSD) with MDD. Based on the literature review, we hypothesized that the individualized status of being a national athlete and the educational awareness of doping would affect moral disengagement in doping and mindfulness among wrestlers in Turkey.

## 2. Methods

### 2.1. Study design and participants

**Figure 1:**
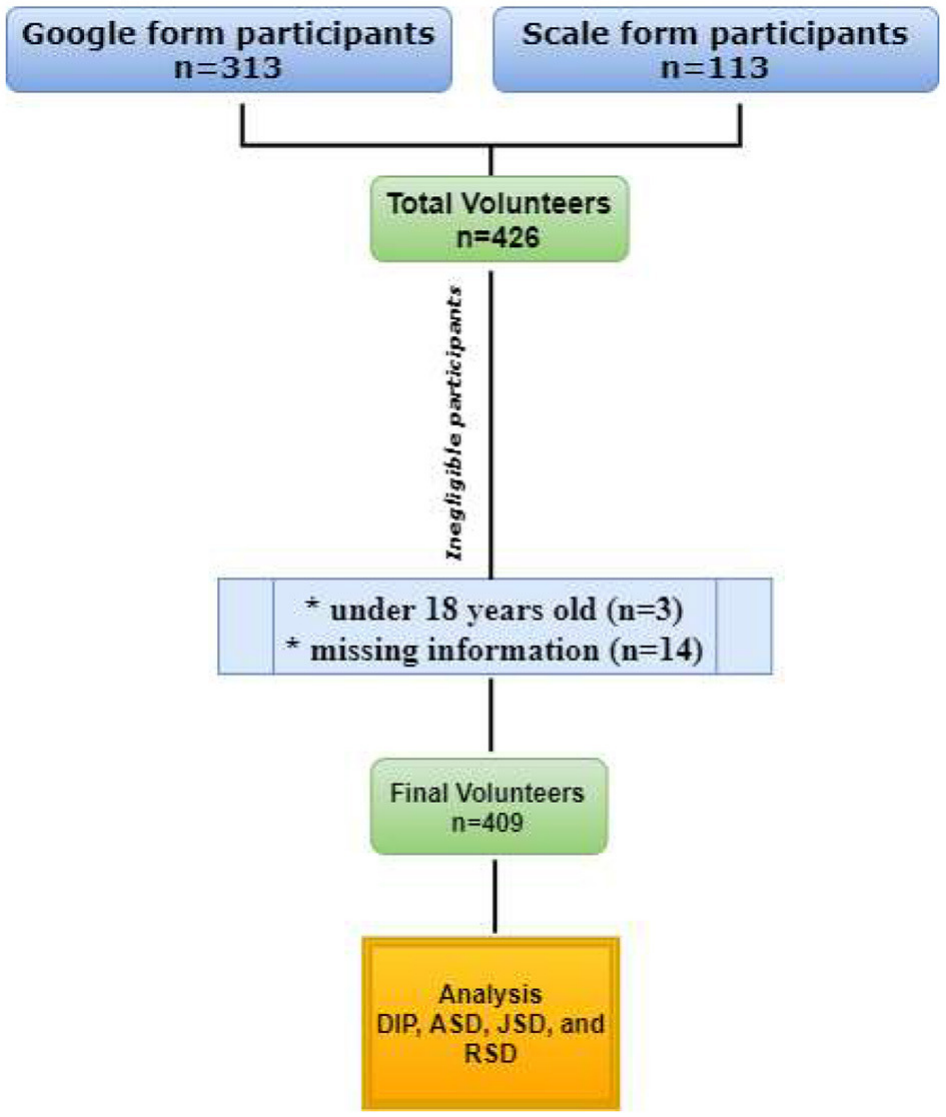
Data collecting procedure.

In this cross-sectional study, the convenience sampling method was conducted on 426 wrestlers who participated in the study (Figure 1). A total of 17 participants were excluded as follows: three participants were under 18 years old, and 14 had missing data. A total of 409 licensed male wrestlers voluntarily agreed to participate in this study and were included in the analysis. Of those, 127 wrestlers (31%) in the sample group were asked to fill in the questionnaire on Google forms, 113 participants were surveyed by handing out a questionnaire, and the remaining participants were reached out *via* social media (Instagram, Facebook, and WhatsApp). The data collection process took place between 26 and 31 July 2022. The convenience sampling method was used while collecting the data. Participants were asked to fill out the form immediately within a maximum of 30 min. Data were collected by reaching out to universities' wrestling teams and sports clubs. Participants were athletes who had received education on doping at the universities.

### 2.2. Procedures

Participation in this study was entirely voluntary. Any participant had the right not to participate or quit the study at any time after participating. The participant's filling in the questions on the measurement tool means that he gave his consent to participate in the research. It was stated that no one should be under pressure while answering the questions, and that the data obtained from the study will be used for research purposes only. This study was conducted in accordance with the tenets of the Declaration of Helsinki. Ethical approval was obtained from the Ethics Committee of Tekirdag Namik Kemal University.

### 2.3. Measurements

#### 2.3.1. Questionnaires

The first part of the questionnaire includes demographic information consisting of doping-related education status and being a national athlete. In the second part, the Mindfulness scale was used, which has been used in previous studies (Thienot et al., 2014; TIngaz, 2020). The Mindfulness scale consists of three dimensions: awareness, judgment, and refocus. Each dimension consists of five items rated on a 6-point Likert-type scale. The judgment dimension of the Mindfulness scale was calculated by reverse coding. In the third part, moral disengagement in the Doping in Sport Scale was used, which was developed by Kavussanu et al. (2016) and adapted by Gürpinar et al. (2019). The scale consists of six statements measured on a Likert scale ranging from 1 (strongly disagree) to 7 (strongly agree).

#### 2.3.2. Data analysis

Kurtosis and skewness values were examined to check whether the data were homogeneous. Descriptive data were reported as mean ± standard deviation (SD) for continuous variables and frequency and percentages for categorical variables. In order to assess the relationship between the variable of interest, Pearson correlation analysis was performed. In the study, the MANOVA test was used to examine the main effect of a national athlete (NA), doping-related education (DRE), and the interaction effect of NA^*^DRE. In order to perform the MANOVA analysis, it was first examined whether the sub-dimensions of the athlete awareness scale and moral disengagement in the Doping in Sport Scale provided the assumption of normal distribution. To verify the normality assumptions of MANOVA, if the sample presents skewness and kurtosis coefficients in the range of ±2, the data are homogeneous, and MANOVA analysis can be performed (Keselman et al., 1998; French et al., 2008; Warne, 2014). In addition, the absence of data below 30 between groups (*n* > 30) meets the assumption of normality (Montgomery et al., 2021; Yağin F. H. et al., 2021). Box's test of equality of covariance matrices was determined as *p* < 0.001. Therefore, Pillai's trace results have been taken into account. Partial eta-squares ($\eta_{p}^{2}$) were calculated to examine the magnitude of the effect between groups.

When Cronbach's alpha reliability analysis was examined, the result was 0.79 in the awareness sub-dimension (ASD), 0.79 in the judgment sub-dimension (JSD), 0.81 in the refocusing sub-dimension (RSD), and 0.86 in moral disengagement in doping (MDD) level. All statistical analyses were conducted using SPSS software (IBM SPSS Statistics version 25; Armonk, NY, USA). Statistical significance was set at alpha level < 0.05. American Psychological Association (APA) 6.0 style was used to report statistical differences (Yağin B. et al., 2021).

## 3. Results

Descriptive statistics of the athletes show that 54.1% of the athletes who have received anti-doping education were NA, and 45.9% were not NA (Table 1).

**Table 1:** Descriptive data of the sample (*n* = 409).

**Variable**		**NA**	**Not NA**
**Doping-related education status**	**Yes**	92 (54.1%)	78 (45.9%)
	**No**	35 (14.7%)	205 (85.3%)

Table 3 shows the results of the MANOVA test for scale scores of the interaction effect of NA and DRE. In the case of being an NA, while there was a significant difference in the ASD and RSD, there was no significance in the MDD and JSD. It has been determined that the awareness levels of NA are significantly higher than those who are not NA. At the same time, it is seen that the awareness levels of the wrestlers who are not NA but who have received training are higher than those who have not received training. In the DRE, while there was a significant difference in the RSD, there was no significant difference in the MDD, JSD, and ASD. In the RSD, it was determined that NA who received doping training scored significantly higher than those who did not receive doping training (Tables 2, 3). When the results of the interaction effect for the status of being an NA and DRE were examined, it was determined that there was a significant difference in the MDD, JSD, ASD, and RSD (*p* < 0.05). According to results, it was determined that the highest effect was on the ASD of being an NA ($\eta_{p}^{2}$ = 0.173).

**Table 2:** Descriptive statistics of MDD, ASD, JSD, and RSD scores for NA and DRE groups.

**National athlete status**	**DRE**	**MDD**	**ASD**	**JSD**	**RSD**
		**M** ±**SD**	**M** ±**SD**	**M** ±**SD**	**M** ±**SD**
NA	Yes	3.850 ± 0.673	5.248 ± 0.623	3.157 ± 0.465	4.939 ± 0.612
	No	4.371 ± 0.986	5.371 ± 0.395	3.394 ± 0.695	5.029 ± 0.623
	Total	3.993 ± 0.803	5.282 ± 0.570	3.222 ± 0.546	4.964 ± 0.614
Non-NA	Yes	4.372 ± 1.059	4.518 ± 1.185	3.413 ± 0.698	4.697 ± 1.065
	No	4.121 ± 1.321	3.965 ± 1.074	3.356 ± 0.395	4.076 ± 0.934
	Total	4.190 ± 1.257	4.118 ± 1.131	3.372 ± 0.497	4.248 ± 1.009
Total	Yes	4.089 ± 0.907	4.913 ± 0.991	3.274 ± 0.595	4.828 ± 0.856
	No	4.158 ± 1.278	4.171 ± 1.120	3.362 ± 0.450	4.216 ± 0.955
	Total	4.129 ± 1.138	4.479 ± 1.128	3.325 ± 0.517	4.470 ± 0.963

NA, national athlete; DRE, doping-related education; MDD, moral disengagement in doping; ASD, awareness sub-dimension; JSD, judgment sub-dimension; RSD, refocusing sub-dimension; M, mean; SD, standard deviation.

**Table 3:** Differences in moral disengagement in doping and athlete mindfulness by the NA and DRE.

Source*		**Type III sum of squares**	**df**	**Mean square**	**F**	**Sig**.	** $\eta_{p}^{2}$ **
**Tests of between-subjects effects**							
NA	MDD^a^	1.291	1	1.291	1.016	0.314	0.003
	ASD^b^	79.860	1	79.860	84.460	**<0.001**	0.173
	JSD^c^	0.831	1	0.831	3.195	0.075	0.008
	RSD^d^	24.931	1	24.931	32.406	**<0.001**	0.074
DRE	MDD	1.284	1	1.284	1.010	0.315	0.002
	ASD	3.229	1	3.229	3.415	0.065	0.008
	JSD	0.572	1	0.572	2.200	0.139	0.005
	RSD	4.942	1	4.942	6.424	**0.012**	0.016
NA* DRE	MDD	10.444	1	10.444	8.218	**0.004**	0.020
	ASD	8.014	1	8.014	8.476	**0.004**	0.020
	JSD	1.519	1	1.519	5.844	**0.016**	0.014
	RSD	8.829	1	8.829	11.476	**0.001**	0.028

^*^MANOVA test; NA, national athlete; DRE, doping-related education; MDD, moral disengagement in doping; ASD, awareness sub-dimension; JSD, judgment sub-dimension; RSD, refocusing sub-dimension;

a R^2^ = 0.026;

b R^2^ = 0.263;

c R^2^ = 0.257;

d R^2^ = 0.170;

$\eta_{p}^{2}$: partial eta-squares; bold font indicates statistical significance.

Table 4 shows that MDD has a low level of negative correlation with ASD and RSD and correlates weakly and positively with JSD. While there is a low level of positive correlation between ASD and JSD, there is a high level of positive correlation between ASD and RSD. On the other hand, there is a moderate positive relationship between JSD and RSD.

**Table 4:** Pearson correlation analysis between sub-dimensions of athlete mindfulness scale and MDD.

	**M ±SD**	**MDD**	**ASD**	**JSD**	**RSD**
DIP	4.12 ± 1.13	1			
ASD	4.47 ± 1.12	−0.126	1		
JSD	3.32 ±0.51	0.140	0.190	1	
RSD	4.47 ± 0.96	−0.041	0.844	0.417	1

M, mean; SD, standard deviation; MDD, moral disengagement in doping; ASD, awareness sub-dimension; JSD, judgment sub-dimension; RSD, refocusing sub-dimension.

## 4. Discussion

This study provides for the first time, to the best of the author's knowledge, information related to anti-doping education and competitive level status and the effect of the latter variables in mindfulness and moral disengagement in doping in Turkish wrestlers' athletes. Descriptive results show a moderate–high level of moral disengagement in doping in wrestlers. In comparison with previous studies, where moral disengagement in doping is analyzed in national-standard-level athletes from other countries (Ring and Kavussanu, 2018; García-Grimau et al., 2022), Turkish athletes present higher levels of moral disengagement, thus becoming more susceptible to doping. Moreover, MDD correlates negatively with the awareness and refocus sub-dimensions of the Mindfulness scale and positively with the judgment sub-dimension. This finding suggests that the more mindful the wrestlers are, the less prone to doping they become. Mental training can not only help to improve athletic performance but also mitigate the possible psychological damage caused by doping in the clean athlete, thus helping the athlete to be less vulnerable to doping.

The deterrence approach in anti-doping is carried out through doping control. Scientific analysis to detect PEDs and substance abuse is continuously improving (Mannocchi et al., 2020). However, doping tests can be seen as costly and may cause negative results when used with new technologies (Trout and Kazlauskas, 2004; Backhouse et al., 2009b). It is important to adopt an educational approach within the sports community and provide anti-doping education not only to the athletes but also to the athlete's support personnel (Goldberg et al., 2000; Hanson, 2009; Mazanov et al., 2011; Barkoukis et al., 2019). Our study shows that the national-level variable has a significant effect on awareness and refocus. However, when the national athlete status and education variable are analyzed together, it has an effect on doping moral disengagement and mindfulness.

WADA can diversify its education program and deterrent planning according to the type of sport to reduce athletes' attitudes toward doping use (Whitaker et al., 2014). Although some studies show that doping education deters attitudes toward doping use (Murofushi et al., 2018; Bayrakdaroglu et al., 2022; Eken and Kafkas, 2022), some studies also show that athletes tend to use doping even though they know about it (Kim and Kim, 2017).

In conclusion, all these results show that education on doping is vital for wrestlers. We recommend increasing awareness, anti-doping education, and mental training among national-standard Turkish wrestlers to prevent them from engaging in doping behaviors.

## 5. Conclusion and limitations

With the ambition to win in sports and pursue performance improvement, athletes may engage voluntarily or unintentionally in doping behavior. To what extent they are aware of this situation is a matter of interest. It is thought that WADA's studies on doping education will reduce the attitude toward doping use. Our study shows a significant effect on the interaction between DRE and being an NA, with moral disengagement in doping and athlete mindfulness sub-dimensions. However, since this effect is low, it should be evaluated with other factors. Especially during the pandemic, the desire to enhance performance may be higher due to social restrictions, the lockdown, and the lack of competitions. Recent research has demonstrated the impact of PEDs use and substance use on public health during the pandemic due to higher stress and psychosocial conditions, which have led to an increase in substance use disorders (Kumar et al., 2022; Negro et al., 2022). In the sporting context, anti-doping integrity issues have arisen alongside the opportunity for stakeholders to develop and implement solutions (Lima et al., 2021). Mindfulness may play a crucial role as a protective factor against the use of PEDs. Further research is needed to better understand the association between mindfulness, doping moral disengagement, and substance abuse in sports.

There are some limitations in this research. The sample distribution revealed that the study included only male respondents, which may influence the generalizability of the finding. The study is a cross-sectional design and provides information about relationships but not causality. A total of 409 male wrestlers were included in the sample group. These wrestlers are required to be over 18 years old, have at least 1 year of licensed wrestling, and should have participated in at least one national competition. In previous studies on wrestlers and WADA's studies, it has been emphasized that anti-doping education is important. Therefore, this study provides valuable and innovative insight with respect to wrestlers who are educated in doping and those who are not. This study on doping-related attitudes and athlete mindfulness may shed light on future studies on sports psychology. In addition, making comparisons with female wrestlers, expanding the sample group, including athletes support personnel (trainers, sports managers, sports psychologists, etc.), and conducting this study in different sports will make a significant contribution to the field of sports psychology.

## Data availability statement

The raw data supporting the conclusions of this article will be made available by the authors, without undue reservation.

## Ethics statement

The study was approved by Tekirdag Namik Kemal University Social and Human Sciences Scientific Research and Publication Ethics Committee (dated 23.06.2022 and numbered 173291). The patients/participants provided their written informed consent to participate in this study.

## Author contributions

EA: conceptualization, methodology, and investigation. HK: software, resources, visualization, and data curation. FY and HK: validation. FY: formal analysis. HK and EA: data curation. EA and MG: writing of original draft preparation. EA, MG, EG-G, MA, and SA-M: writing of review and editing. MA: funding acquisition. All authors contributed to the article and approved the submitted version.
